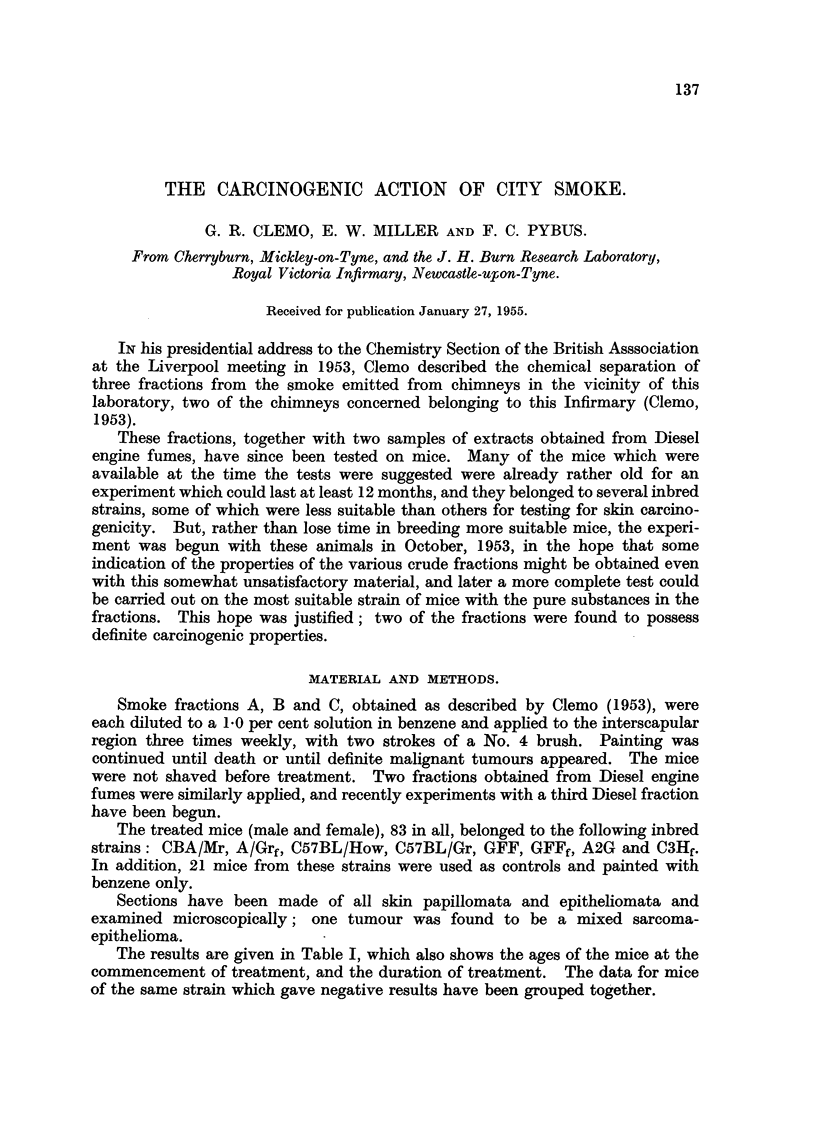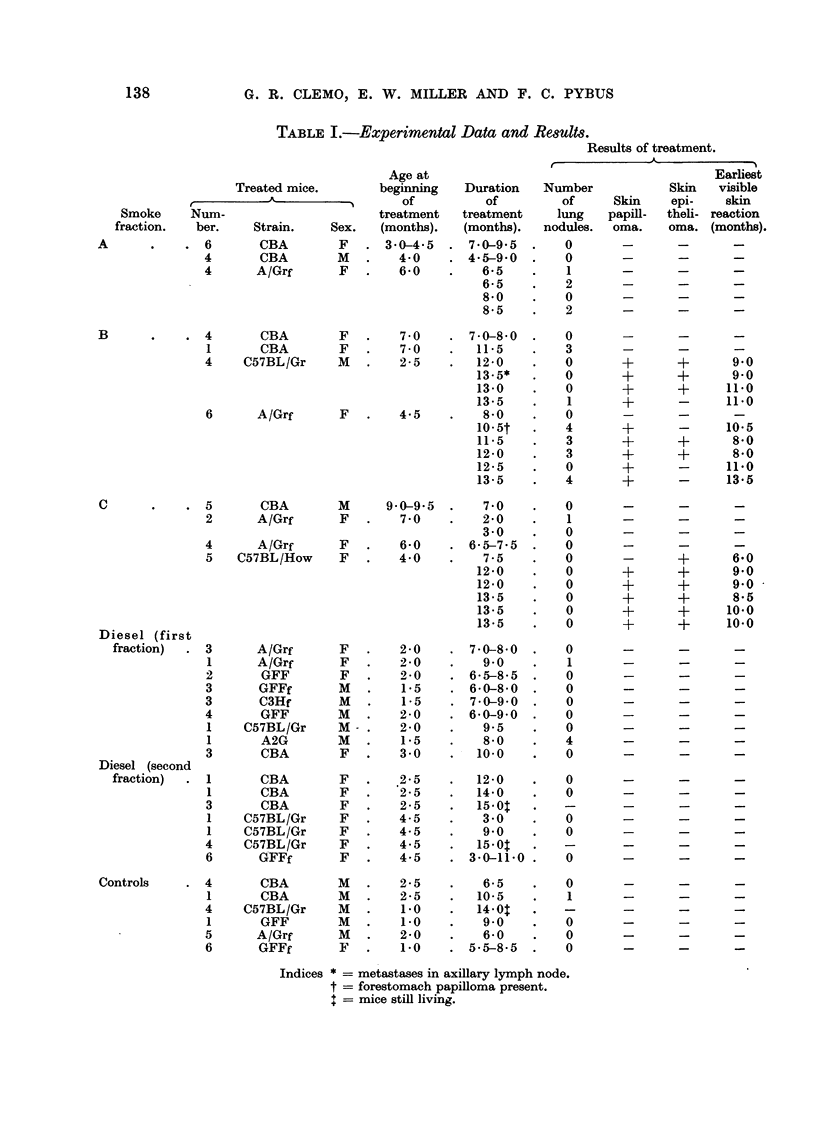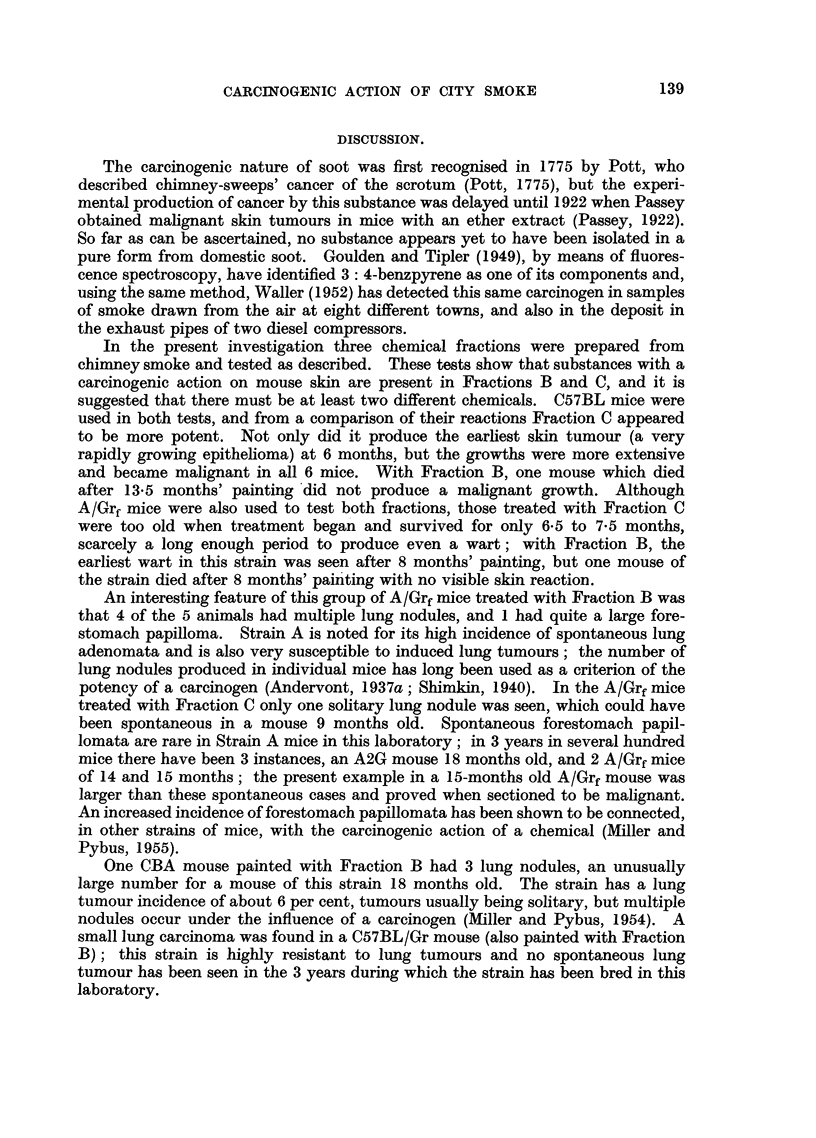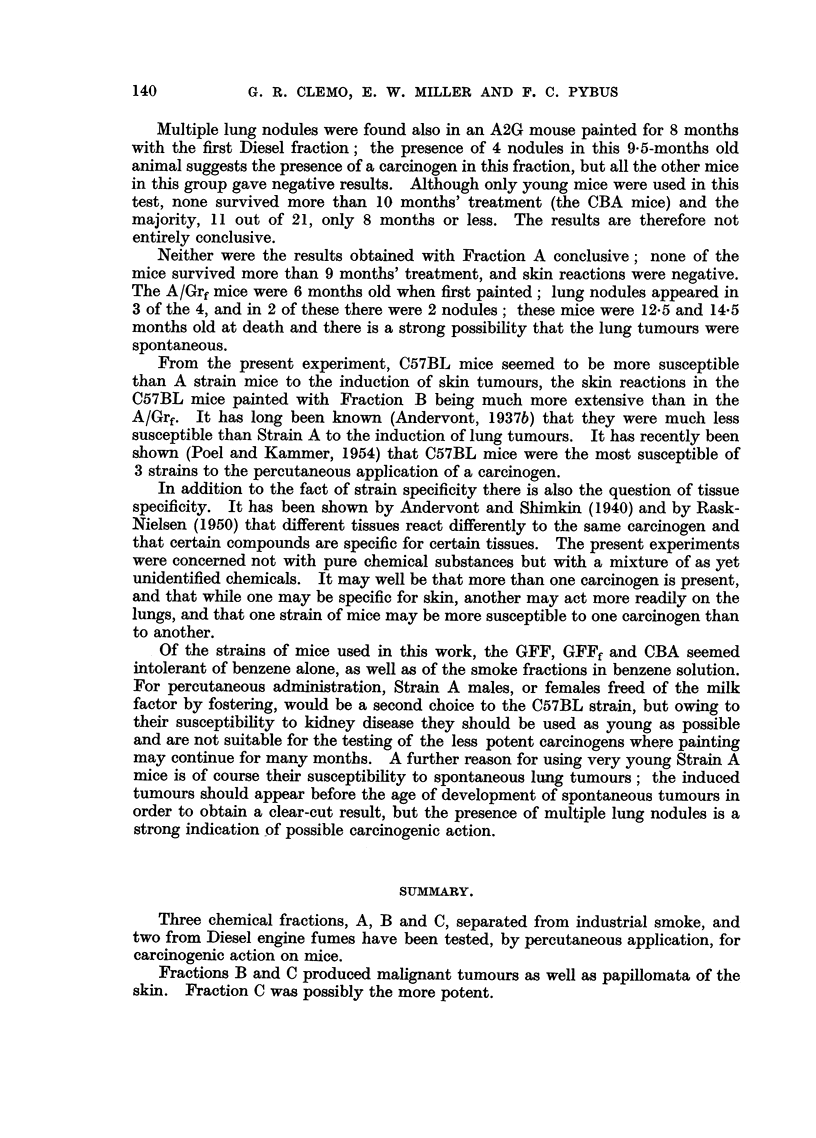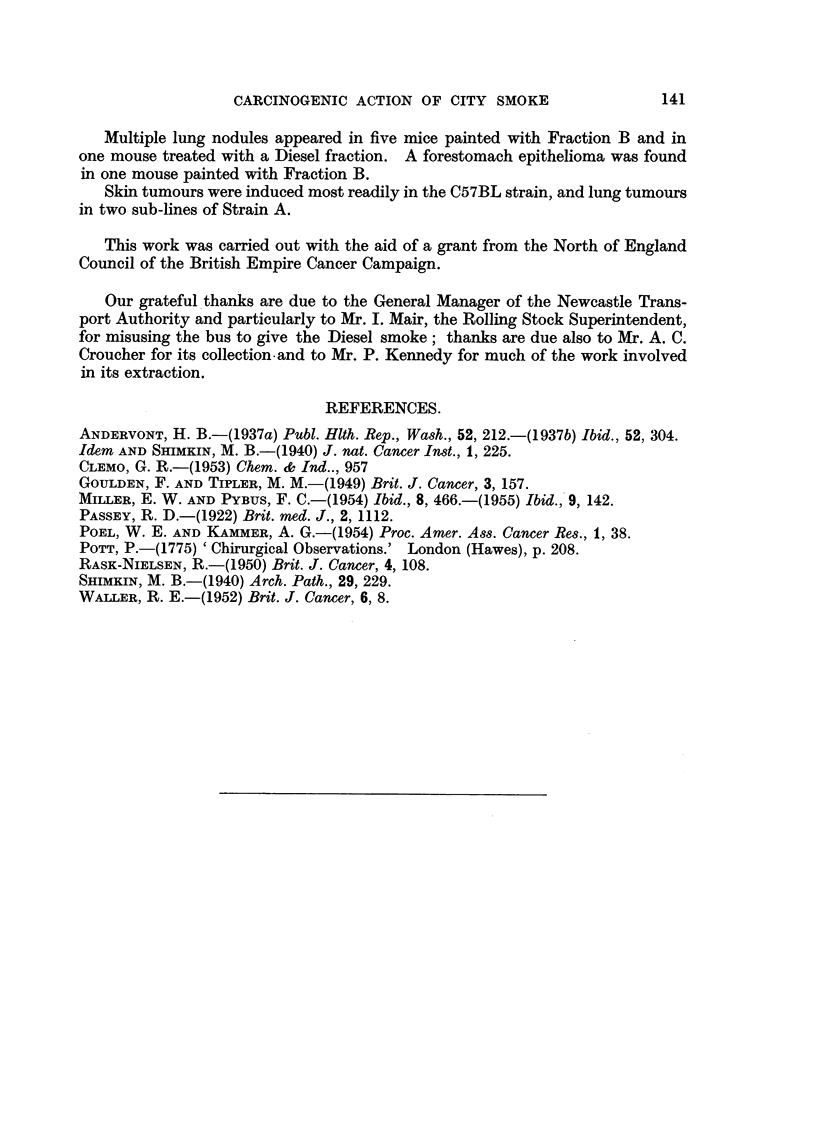# The Carcinogenic Action of City Smoke

**DOI:** 10.1038/bjc.1955.10

**Published:** 1955-03

**Authors:** G. R. Clemo, E. W. Miller, F. C. Pybus


### THE CARCINOGENIC ACTION OF CITY SMOKE.

G. R. CLEMO, E. W. MILLER AND F. C. PYBUS.

*From Cherryburn, Mickley-on-Tyne, and the J. H. Burn Research Laboratory, Royal Victoria Infirmary, Newcastle-upon-Tyne.*

Received for publication January 27, 1955.

IN his presidential address to the Chemistry Section of the British Asssociation at the Liverpool meeting in 1953, Clemo described the chemical separation of three fractions from the smoke emitted from chimneys in the vicinity of this laboratory, two of the chimneys concerned belonging to this Infirmary (Clemo, 1953).

These fractions, together with two samples of extracts obtained from Diesel engine fumes, have since been tested on mice. Many of the mice which were available at the time the tests were suggested were already rather old for an experiment which could last at least 12 months, and they belonged to several inbred strains, some of which were less suitable than others for testing for skin carcinogenicity. But, rather than lose time in breeding more suitable mice, the experiment was begun with these animals in October, 1953, in the hope that some indication of the properties of the various crude fractions might be obtained even with this somewhat unsatisfactory material, and later a more complete test could be carried out on the most suitable strain of mice with the pure substances in the fractions. This hope was justified; two of the fractions were found to possess definite carcinogenic properties.

### MATERIAL AND METHODS.

Smoke fractions A, B and C, obtained as described by Clemo (1953), were each diluted to a 1·0 per cent solution in benzene and applied to the interscapular region three times weekly, with two strokes of a No. 4 brush. Painting was continued until death or until definite malignant tumours appeared. The mice were not shaved before treatment. Two fractions obtained from Diesel engine fumes were similarly applied, and recently experiments with a third Diesel fraction have been begun.

The treated mice (male and female), 83 in all, belonged to the following inbred strains: CBA/Mr, A/$Gr_f$, C57BL/How, C57BL/Gr, GFF, $GFF_f$, A2G and $C3H_f$. In addition, 21 mice from these strains were used as controls and painted with benzene only.

Sections have been made of all skin papillomata and epitheliomata and examined microscopically; one tumour was found to be a mixed sarcoma-epithelioma.

The results are given in Table I, which also shows the ages of the mice at the commencement of treatment, and the duration of treatment. The data for mice of the same strain which gave negative results have been grouped together.

TABLE I.—*Experimental Data and Results.*

| Smoke fraction. | Treated mice. | | | Age at beginning of treatment (months). | Duration of treatment (months). | Results of treatment. | | | |
|---|---|---|---|---|---|---|---|---|---|
| | Number. | Strain. | Sex. | | | Number of lung nodules. | Skin papilloma. | Skin epithelioma. | Earliest visible skin reaction (months). |
| A | 6 | CBA | F | 3·0–4·5 | 7·0–9·5 | 0 | — | — | — |
| | 4 | CBA | M | 4·0 | 4·5–9·0 | 0 | — | — | — |
| | 4 | A/Grf | F | 6·0 | 6·5 | 1 | — | — | — |
| | | | | | 6·5 | 2 | — | — | — |
| | | | | | 8·0 | 0 | — | — | — |
| | | | | | 8·5 | 2 | — | — | — |
| B | 4 | CBA | F | 7·0 | 7·0–8·0 | 0 | — | — | — |
| | 1 | CBA | F | 7·0 | 11·5 | 3 | — | — | — |
| | 4 | C57BL/Gr | M | 2·5 | 12·0 | 0 | + | + | 9·0 |
| | | | | | 13·5* | 0 | + | + | 9·0 |
| | | | | | 13·0 | 0 | + | + | 11·0 |
| | | | | | 13·5 | 1 | + | — | 11·0 |
| | 6 | A/Grf | F | 4·5 | 8·0 | 0 | — | — | — |
| | | | | | 10·5† | 4 | + | — | 10·5 |
| | | | | | 11·5 | 3 | + | + | 8·0 |
| | | | | | 12·0 | 3 | + | + | 8·0 |
| | | | | | 12·5 | 0 | + | — | 11·0 |
| | | | | | 13·5 | 4 | + | — | 13·5 |
| C | 5 | CBA | M | 9·0–9·5 | 7·0 | 0 | — | — | — |
| | 2 | A/Grf | F | 7·0 | 2·0 | 1 | — | — | — |
| | | | | | 3·0 | 0 | — | — | — |
| | 4 | A/Grf | F | 6·0 | 6·5–7·5 | 0 | — | — | — |
| | 5 | C57BL/How | F | 4·0 | 7·5 | 0 | — | + | 6·0 |
| | | | | | 12·0 | 0 | + | + | 9·0 |
| | | | | | 12·0 | 0 | + | + | 9·0 |
| | | | | | 13·5 | 0 | + | + | 8·5 |
| | | | | | 13·5 | 0 | + | + | 10·0 |
| | | | | | 13·5 | 0 | + | + | 10·0 |
| Diesel (first fraction) | 3 | A/Grf | F | 2·0 | 7·0–8·0 | 0 | — | — | — |
| | 1 | A/Grf | F | 2·0 | 9·0 | 1 | — | — | — |
| | 2 | GFF | F | 2·0 | 6·5–8·5 | 0 | — | — | — |
| | 3 | GFFf | M | 1·5 | 6·0–8·0 | 0 | — | — | — |
| | 3 | C3Hf | M | 1·5 | 7·0–9·0 | 0 | — | — | — |
| | 4 | GFF | M | 2·0 | 6·0–9·0 | 0 | — | — | — |
| | 1 | C57BL/Gr | M | 2·0 | 9·5 | 0 | — | — | — |
| | 1 | A2G | M | 1·5 | 8·0 | 4 | — | — | — |
| | 3 | CBA | F | 3·0 | 10·0 | 0 | — | — | — |
| Diesel (second fraction) | 1 | CBA | F | 2·5 | 12·0 | 0 | — | — | — |
| | 1 | CBA | F | 2·5 | 14·0 | 0 | — | — | — |
| | 3 | CBA | F | 2·5 | 15·0‡ | — | — | — | — |
| | 1 | C57BL/Gr | F | 4·5 | 3·0 | 0 | — | — | — |
| | 1 | C57BL/Gr | F | 4·5 | 9·0 | 0 | — | — | — |
| | 4 | C57BL/Gr | F | 4·5 | 15·0‡ | — | — | — | — |
| | 6 | GFFf | F | 4·5 | 3·0–11·0 | 0 | — | — | — |
| Controls | 4 | CBA | M | 2·5 | 6·5 | 0 | — | — | — |
| | 1 | CBA | M | 2·5 | 10·5 | 1 | — | — | — |
| | 4 | C57BL/Gr | M | 1·0 | 14·0‡ | — | — | — | — |
| | 1 | GFF | M | 1·0 | 9·0 | 0 | — | — | — |
| | 5 | A/Grf | M | 2·0 | 6·0 | 0 | — | — | — |
| | 6 | GFFf | F | 1·0 | 5·5–8·5 | 0 | — | — | — |

Indices * = metastases in axillary lymph node.
† = forestomach papilloma present.
‡ = mice still living.

### DISCUSSION.

The carcinogenic nature of soot was first recognised in 1775 by Pott, who described chimney-sweeps' cancer of the scrotum (Pott, 1775), but the experimental production of cancer by this substance was delayed until 1922 when Passey obtained malignant skin tumours in mice with an ether extract (Passey, 1922). So far as can be ascertained, no substance appears yet to have been isolated in a pure form from domestic soot. Goulden and Tipler (1949), by means of fluorescence spectroscopy, have identified 3 : 4-benzpyrene as one of its components and, using the same method, Waller (1952) has detected this same carcinogen in samples of smoke drawn from the air at eight different towns, and also in the deposit in the exhaust pipes of two diesel compressors.

In the present investigation three chemical fractions were prepared from chimney smoke and tested as described. These tests show that substances with a carcinogenic action on mouse skin are present in Fractions B and C, and it is suggested that there must be at least two different chemicals. C57BL mice were used in both tests, and from a comparison of their reactions Fraction C appeared to be more potent. Not only did it produce the earliest skin tumour (a very rapidly growing epithelioma) at 6 months, but the growths were more extensive and became malignant in all 6 mice. With Fraction B, one mouse which died after 13·5 months' painting did not produce a malignant growth. Although $A/Gr_f$ mice were also used to test both fractions, those treated with Fraction C were too old when treatment began and survived for only 6·5 to 7·5 months, scarcely a long enough period to produce even a wart; with Fraction B, the earliest wart in this strain was seen after 8 months' painting, but one mouse of the strain died after 8 months' painting with no visible skin reaction.

An interesting feature of this group of $A/Gr_f$ mice treated with Fraction B was that 4 of the 5 animals had multiple lung nodules, and 1 had quite a large fore-stomach papilloma. Strain A is noted for its high incidence of spontaneous lung adenomata and is also very susceptible to induced lung tumours; the number of lung nodules produced in individual mice has long been used as a criterion of the potency of a carcinogen (Andervont, 1937*a*; Shimkin, 1940). In the $A/Gr_f$ mice treated with Fraction C only one solitary lung nodule was seen, which could have been spontaneous in a mouse 9 months old. Spontaneous forestomach papillomata are rare in Strain A mice in this laboratory; in 3 years in several hundred mice there have been 3 instances, an A2G mouse 18 months old, and 2 $A/Gr_f$ mice of 14 and 15 months; the present example in a 15-months old $A/Gr_f$ mouse was larger than these spontaneous cases and proved when sectioned to be malignant. An increased incidence of forestomach papillomata has been shown to be connected, in other strains of mice, with the carcinogenic action of a chemical (Miller and Pybus, 1955).

One CBA mouse painted with Fraction B had 3 lung nodules, an unusually large number for a mouse of this strain 18 months old. The strain has a lung tumour incidence of about 6 per cent, tumours usually being solitary, but multiple nodules occur under the influence of a carcinogen (Miller and Pybus, 1954). A small lung carcinoma was found in a C57BL/Gr mouse (also painted with Fraction B); this strain is highly resistant to lung tumours and no spontaneous lung tumour has been seen in the 3 years during which the strain has been bred in this laboratory.

Multiple lung nodules were found also in an A2G mouse painted for 8 months with the first Diesel fraction; the presence of 4 nodules in this 9·5-months old animal suggests the presence of a carcinogen in this fraction, but all the other mice in this group gave negative results. Although only young mice were used in this test, none survived more than 10 months' treatment (the CBA mice) and the majority, 11 out of 21, only 8 months or less. The results are therefore not entirely conclusive.

Neither were the results obtained with Fraction A conclusive; none of the mice survived more than 9 months' treatment, and skin reactions were negative. The $A/Gr_f$ mice were 6 months old when first painted; lung nodules appeared in 3 of the 4, and in 2 of these there were 2 nodules; these mice were 12·5 and 14·5 months old at death and there is a strong possibility that the lung tumours were spontaneous.

From the present experiment, C57BL mice seemed to be more susceptible than A strain mice to the induction of skin tumours, the skin reactions in the C57BL mice painted with Fraction B being much more extensive than in the $A/Gr_f$. It has long been known (Andervont, 1937*b*) that they were much less susceptible than Strain A to the induction of lung tumours. It has recently been shown (Poel and Kammer, 1954) that C57BL mice were the most susceptible of 3 strains to the percutaneous application of a carcinogen.

In addition to the fact of strain specificity there is also the question of tissue specificity. It has been shown by Andervont and Shimkin (1940) and by Rask-Nielsen (1950) that different tissues react differently to the same carcinogen and that certain compounds are specific for certain tissues. The present experiments were concerned not with pure chemical substances but with a mixture of as yet unidentified chemicals. It may well be that more than one carcinogen is present, and that while one may be specific for skin, another may act more readily on the lungs, and that one strain of mice may be more susceptible to one carcinogen than to another.

Of the strains of mice used in this work, the GFF, $GFF_f$ and CBA seemed intolerant of benzene alone, as well as of the smoke fractions in benzene solution. For percutaneous administration, Strain A males, or females freed of the milk factor by fostering, would be a second choice to the C57BL strain, but owing to their susceptibility to kidney disease they should be used as young as possible and are not suitable for the testing of the less potent carcinogens where painting may continue for many months. A further reason for using very young Strain A mice is of course their susceptibility to spontaneous lung tumours; the induced tumours should appear before the age of development of spontaneous tumours in order to obtain a clear-cut result, but the presence of multiple lung nodules is a strong indication of possible carcinogenic action.

### SUMMARY.

Three chemical fractions, A, B and C, separated from industrial smoke, and two from Diesel engine fumes have been tested, by percutaneous application, for carcinogenic action on mice.

Fractions B and C produced malignant tumours as well as papillomata of the skin. Fraction C was possibly the more potent.

Multiple lung nodules appeared in five mice painted with Fraction B and in one mouse treated with a Diesel fraction. A forestomach epithelioma was found in one mouse painted with Fraction B.

Skin tumours were induced most readily in the C57BL strain, and lung tumours in two sub-lines of Strain A.

This work was carried out with the aid of a grant from the North of England Council of the British Empire Cancer Campaign.

Our grateful thanks are due to the General Manager of the Newcastle Transport Authority and particularly to Mr. I. Mair, the Rolling Stock Superintendent, for misusing the bus to give the Diesel smoke ; thanks are due also to Mr. A. C. Croucher for its collection and to Mr. P. Kennedy for much of the work involved in its extraction.

### REFERENCES.

ANDERVONT, H. B.—(1937*a*) *Publ. Hlth. Rep., Wash.*, **52**, 212.—(1937*b*) *Ibid.*, **52**, 304.
*Idem* AND SHIMKIN, M. B.—(1940) *J. nat. Cancer Inst.*, **1**, 225.
CLEMO, G. R.—(1953) *Chem. & Ind..*, 957
GOULDEN, F. AND TIPLER, M. M.—(1949) *Brit. J. Cancer*, **3**, 157.
MILLER, E. W. AND PYBUS, F. C.—(1954) *Ibid.*, **8**, 466.—(1955) *Ibid.*, **9**, 142.
PASSEY, R. D.—(1922) *Brit. med. J.*, **2**, 1112.
POEL, W. E. AND KAMMER, A. G.—(1954) *Proc. Amer. Ass. Cancer Res.*, **1**, 38.
POTT, P.—(1775) ‘ Chirurgical Observations.’ London (Hawes), p. 208.
RASK-NIELSEN, R.—(1950) *Brit. J. Cancer*, **4**, 108.
SHIMKIN, M. B.—(1940) *Arch. Path.*, **29**, 229.
WALLER, R. E.—(1952) *Brit. J. Cancer*, **6**, 8.